# Rheumatoid Arthritis in Agricultural Health Study Spouses: Associations with Pesticides and Other Farm Exposures

**DOI:** 10.1289/EHP129

**Published:** 2016-06-10

**Authors:** Christine G. Parks, Jane A. Hoppin, Anneclaire J. De Roos, Karen H. Costenbader, Michael C. Alavanja, Dale P. Sandler

**Affiliations:** 1 Epidemiology Branch, National Institute of Environmental Health Sciences, National Institutes of Health (NIH), Department of Health and Human Services (DHHS), Research Triangle Park, North Carolina, USA; 2 Center for Human Health and the Environment, Department of Biological Sciences, North Carolina State University, Raleigh, North Carolina, USA; 3 Department of Environmental and Occupational Health, Drexel University School of Public Health, Philadelphia, Pennsylvania, USA; 4 Brigham and Women’s Hospital, Harvard Medical School, Boston, Massachusetts, USA; 5 Division of Cancer Epidemiology and Genetics, National Cancer Institute, NIH, DHHS, Bethesda, Maryland, USA

## Abstract

**Background:**

Farming has been associated with rheumatoid arthritis (RA), but the role of pesticides is not known.

**Objectives:**

We examined associations between RA and pesticides or other agricultural exposures among female spouses of licensed pesticide applicators in the Agricultural Health Study.

**Methods:**

Women were enrolled between 1993 and 1997 and followed through 2010. Cases (n = 275 total, 132 incident), confirmed by a physician or by self-reported use of disease modifying antirheumatic drugs, were compared with noncases (n = 24,018). Odds ratios (OR) and 95% confidence intervals (CI) were estimated using logistic regression models adjusted for age, state, and smoking pack-years.

**Results:**

Overall, women with RA were somewhat more likely to have reported lifetime use of any specific pesticide versus no pesticides (OR = 1.4; 95% CI: 1.0, 1.6). Of the 15 pesticides examined, maneb/mancozeb (OR = 3.3; 95% CI: 1.5, 7.1) and glyphosate (OR = 1.4; 95% CI: 1.0, 2.1) were associated with incident RA compared with no pesticide use. An elevated, but non-statistically significant association with incident RA was seen for DDT (OR = 1.9; 95% CI: 0.97, 3.6). Incident RA was also associated with the application of chemical fertilizers (OR = 1.7; 95% CI: 1.1, 2.7) and cleaning with solvents (OR = 1.6; 95% CI: 1.1, 2.4), but inversely associated with lifetime livestock exposure as a child and adult (OR = 0.48; 95% CI: 0.24, 0.97) compared with no livestock exposure.

**Conclusions:**

Our results suggest that specific agricultural pesticides, solvents, and chemical fertilizers may increase the risk of RA in women, while exposures involving animal contact may be protective.

**Citation:**

Parks CG, Hoppin JA, De Roos AJ, Costenbader KH, Alavanja MC, Sandler DP. 2016. Rheumatoid arthritis in Agricultural Health Study spouses: associations with pesticides and other farm exposures. Environ Health Perspect 124:1728–1734; http://dx.doi.org/10.1289/EHP129

## Introduction

Rheumatoid arthritis (RA) is a systemic autoimmune disease characterized by joint-specific and systemic inflammation that affects 2% of the U.S. population who are more than 60 years old, with higher rates among women (Rasch et al. 2003). Established environmental risk factors include crystalline silica dust and smoking (Miller et al. 2012). Farming occupation has also been associated with RA (Gold et al. 2007; Khuder et al. 2002; Lee et al. 2002; Levêque-Morlais et al. 2015; Li et al. 2008; Lundberg et al. 1994; Milham 1988; Olsson et al. 2000). Exposure to pesticides is a commonly hypothesized explanation for this association, and toxicology data suggest complex effects of specific pesticides on the immune system (Holsapple 2002; Luebke et al. 2004). Earlier studies suggested pesticide use was modestly, but non-statistically significantly associated with RA (Khuder et al. 2002; Lundberg et al. 1994). More recent findings from a study of incident RA in Sweden showed inconsistent associations with occupational pesticides among men and women (Olsson et al. 2004), while a 24-state study in the United States showed a statistically significant association of RA mortality with pesticide exposure assessed by a job-exposure matrix (Gold et al. 2007).

Few studies have investigated specific pesticides and RA. In the Women’s Health Initiative, self-reported use of residential insecticides was associated with risk of RA or a related autoimmune disease, systemic lupus erythematosus (SLE). The highest risks were observed for women who reported personally mixing or applying insecticides, especially if they had ever lived or worked on a farm (Parks et al. 2011). Agricultural settings confer a variety of other potentially immune-modulating exposures that may be associated with RA and systemic autoimmune diseases, such as sunlight, inorganic dusts, and endotoxins (Arkema et al. 2013; Hou et al. 2013; Parks et al. 2014). Limited evidence suggests RA may be associated with crop but not livestock farming (Gold et al. 2007; Lee et al. 2002), though findings are inconsistent (Olsson et al. 2004; Reckner Olsson et al. 2001). One study found SLE was inversely associated with childhood livestock exposure, especially if exposure continued in adulthood (Parks et al. 2008). Together, these findings suggest a potential protective role of early and ongoing immune modulating microbial exposures (Rook 2012).

The Agricultural Health Study (AHS) is a longitudinal cohort study of licensed pesticide applicators and their spouses. In a previous study of RA in AHS women, suggestive (but non-statistically significant) associations were seen with use of any pesticides and a few specific pesticides (De Roos et al. 2005). Here we extend this work to a larger sample of incident cases, examining associations with pesticides and other exposures, and exploring the potential modifying effects of growing up on a farm.

## Methods

The AHS is a prospective cohort of licensed pesticide applicators and their spouses in Iowa (IA) and North Carolina (NC) (Alavanja et al. 1996). Private pesticide applicators (farmers) applying for certification to use restricted-use pesticides were enrolled between 1993 and 1997 (Phase 1). The current study sample was drawn from female spouses of private applicators. The present study included exposures and disease diagnoses reported at Phase 1, and diagnoses reported in two structured follow-up interviews (Phase 2, 1998–2003; Phase 3, 2005–2010; see https://aghealth.nih.gov/collaboration/questionnaires.html). Study procedures were approved by the Institutional Review Boards of the National Institutes of Health and its contractors. Participants implied informed consent by returning the enrollment questionnaires and participating in the telephone interview.

### Case Identification and Classification

On the enrollment questionnaire, participants self-reported doctor diagnosis of RA and age at diagnosis. Additional data were collected to confirm and validate clinical RA cases, because self-reported information alone is often nonspecific (Walitt et al. 2008). The previous AHS/RA analysis used physician validated cases [*n* = 135 cases (De Roos et al. 2005)], most of whom were prevalent (i.e., reported at Phase 1). In an updated protocol, we sought to confirm new incident cases reported in Phases 2 and 3 and developed an algorithm using screener data to identify probable cases being treated for RA.

As in the prior study (De Roos et al. 2005), we screened eligible potential cases by telephone to confirm their self-reported RA diagnosis. Using a questionnaire developed by two of the authors (C.G.P. and K.H.C.), self-confirmed cases were asked about current medications used for RA or those used in the past, including disease-modifying antirheumatic drugs (DMARDs) and questions about symptoms, and clinical testing results reflecting standard diagnostic protocols (Kasturi et al. 2014). Because RA, SLE, and related connective tissue diseases (CTD) can overlap and differential diagnoses may be difficult, we also screened other reported CTD. Women who could not be reached by phone were mailed a brief questionnaire including a subset of screener questions on diagnoses and medications. For those unable to complete the screening questionnaire due to illness or death, we adapted questionnaires to collect data from proxies. For cases providing written consent, we contacted physicians to validate disease status and complete symptom and medication checklists.

Data from the screening questionnaire and validation were reviewed for internal consistency by one of the authors (C.G.P.). Cases supported by physician data were considered to be confirmed, and all others reporting the use of DMARDs were considered as probable cases. Cases with conflicting data were adjudicated by a rheumatologist (K.H.C.), who also reviewed a random sample of 15% of confirmed and 10% of probable cases. For sensitivity analyses, we also identified possible cases who took corticosteroids, but not DMARDs, for RA.

### Study Sample

Of the 32,126 enrolled female spouses, 5,434 (17%) did not complete either of the follow-up questionnaires (Phases 2 and 3) and 14 had missing responses on RA on all three surveys (see Figure S1). Of the remaining 26,678, we excluded 56 who reported RA diagnosed before age 20, along with 213 who were missing either age at diagnosis or Phase 2 or 3 data on RA, and 123 women who reported RA at Phase 1, but were ineligible for screening in the prior study for various reasons (e.g., more than 10 years since diagnosis, no self-reported symptoms or testing at baseline) (De Roos et al. 2005). We excluded 676 who self-refuted an earlier report of RA at Phase 2 or 3, leaving 25,610. Of these, 1,192 were considered eligible for RA screening (including 195 who reported another CTD, but not RA). Most were screened by telephone and 46 by mail. Proxy respondents completed 22 screening questionnaires. A total of 131 could not be reached or declined participation, and 81 did not provide information on RA. Of those who were screened for RA, 305 (39%) refuted their diagnosis, and 41 (5%) were refuted by a physician.

Validation data was obtained for a total of 195 self-confirmed cases, and 154 (79%) were confirmed as RA by a physician. Of those screened due to reported SLE or Sjögren’s Syndrome (*n* = 195), we identified 7 new self-reported RA cases in the screening interview, and 4 of these were included in the analysis (1 confirmed and 3 probable incident). Excluding 2 cases and 400 noncases who were missing covariate data, the analysis sample included 24,018 noncases who never reported RA (or SLE and Sjögrens) and 275 confirmed or probable RA cases (132 incident).

Compared to physician-confirmed cases, probable cases were more likely to be from NC (46% vs. 31%) and to be diagnosed since the year 2000 (43% vs. 11%) (see Table S1). A similar proportion were diagnosed or seen by a rheumatologist (91%) and most (75–81%) reported a positive rheumatoid factor (RF). Possible cases, who only reported steroid use for RA, were more likely to be from NC (55%) and less likely to report being tested for RF compared to all other case groups. Given known variation in care by geographic area and physician-type (Schmajuk et al. 2011; Ward 1999; Yazdany et al. 2014), we included some of these possible RA cases in sensitivity analyses, as described below.

### Exposures and Covariates

Baseline questionnaires (see https://aghealth.nih.gov/collaboration/questionnaires.html) included a lifetime and childhood residential farm history (i.e., “Altogether, how many years have you lived or worked on a farm?” and “Before age 18, did you live at least half of your life on a farm?”). Participants were asked whether they ever personally mixed or applied any pesticides during their lifetime, years and days per year mixed or applied, percent time mixed or applied (none, < 50%, ≥ 50%), and were then asked about the use of 50 specified pesticides, by name and type (e.g., herbicides, insecticides). Women were also asked about non-specific household pesticide use. These data on specific agricultural pesticide use and household pesticide use were combined as: no pesticide use (33%), any specified pesticide use (54%), or no specified pesticides reported or residential use only (4% and 9%, respectively). Other questions included farm tasks (e.g., driving tractors, cleaning with solvents at least monthly) in the past year and past growing season (i.e., worked in the fields, applied fertilizers, had contact with animals), and hours per day spent in the sun in the past growing season and 10 years prior. On the Phase 3 questionnaire, women were asked about childhood contact with farm animals.

Covariate data included age, state, race/ethnicity, education, and smoking pack-years. Body mass index and baseline menopause status were not associated with RA after accounting for age. In a multivariable model, race/ethnicity and education were not significant predictors of RA, and so were not included in the final models. In a subset, data on menopause status at diagnosis, birth control pills, and hormone replacement were available; these were not confounders and not included in the final models.

### Statistical Analyses

For lifetime pesticide use and specific pesticides, we examined associations with RA overall and incident RA, including both confirmed and probable cases. For recent tasks and exposures (i.e., in the past year or growing season), we limited the analyses to incident RA. We estimated odds ratios (OR) and 95% confidence intervals (CI) by logistic regression, adjusting for age, state, and pack-years smoking. Models were limited to exposures with at least 5 exposed cases. Results highlighted in the text are statistically significant at *p* < 0.05 unless otherwise noted. Based on our prior hypotheses, we explored heterogeneity through models stratified by childhood farm residence and childhood livestock exposure for the subset with available data, and for descriptive purposes stratified by state. We tested multiplicative interactions when stratified effect estimates appeared substantially different. All analyses were performed using SAS (version 9.3; SAS Institute Inc.)

Because data were missing for 25% of women who reported any lifetime pesticide use on the four overall pesticide dose-related variables (i.e., years used, days per year, percent of the time applied, and percent of the time mixed), we used Proc MI to impute missing values. Factors included in the imputation model included age, state, education, smoking pack-years, case or non-case status, doctor’s visit in the past year, farm and family size, years lived/worked on a farm, number of specific pesticides reported, field work in the past growing season, ever and years worked off the farm. After 10 iterations, we used Proc MIANANYZE to perform the analyses.

Sensitivity analyses excluded cases diagnosed the first 2 years after enrollment to reduce the influence of pre-clinical symptoms; we added “possible” cases using corticosteroids only and restricted this expanded case group (confirmed, probable, or possible) to those reporting a positive RF test. We conducted the analyses using the following files from the AHS database: AHSREL201304.00 (demographics release, 2 May 2013); P1REL090600 (Phase 1 release, 31 July 2009); P2REL201007.00 (Phase 2 release, 30 July 2010), and P3REL1000.00 (Phase 3, 18 December 2010). Original data is stored on secure servers by the AHS coordinating center in Westat, Rockville, MD, on behalf of the Agricultural Health Study (https://aghealth.nih.gov/), National Cancer Institute (NCI), Rockville, MD and National Institute of Environmental Health Sciences (NIEHS).

## Results

Sample characteristics are shown in Table 1. Cases were older, and adjusting for age, RA was associated with pack-years smoked, and inversely associated with living in IA. After adjusting for age, no associations were seen with growing up on a farm or years lived on a farm.

**Table 1:** Characteristics of RA cases and noncases among female spouses in the Agricultural Health Study, 1993–2010.

Characteristic	Comparison *n *= 24,018 *n* (%)	All RA cases		Incident cases	
		Case *n *= 275 *n* (%)	OR (95% CI)	Case *n *= 132 *n* (%)	OR (95% CI)
Age at enrollment
< 40 years old	7,548 (31)	35 (13)	1.0 (referent)	21 (16)	1.0 (referent)
40–49 years old	7,178 (30)	65 (24)	2.0 (1.3, 2.9)	37 (28)	1.9 (1.1, 3.2)
50–59 years old	5,726 (24)	107 (39)	4.0 (2.7, 5.9)	47 (36)	2.8 (1.8, 4.9)
≥ 60 years old	3,566 (15)	68 (25)	4.1 (2.7, 6.2)	27 (20)	2.9 (1.5, 4.8)
State
North Carolina	6,929 (29)	105 (38)	1.0 (referent)	46 (35)	1.0 (referent)
Iowa	17,089 (71)	170 (62)	0.75 (0.58, 0.96)	86 (65)	0.83 (0.58, 1.2)
Race
White	23,089 (98)	262 (97)	1.0 (referent)	128 (98)	1.0 (referent)
Nonwhite	523 (2)	9 (3)	1.4 (0.71, 2.7)	2 (2)	0.67 (0.16, 2.7)
Education
≤ High school	9,155 (43)	119 (49)	1.0 (referent)	50 (43)	1.0 (referent)
> High school	11,921 (57)	122 (51)	1.0 (0.80, 1.3)	67 (57)	1.3 (0.87, 1.9)
Smoking pack-years
None	18,027 (75)	200 (73)	1.0 (referent)	93 (70)	1.0 (referent)
< 5 pack-years	2,639 (11)	22 (8)	0.85 (0.55, 1.3)	14 (11)	1.1 (0.64, 2.0)
5–18 pack-years	1,964 (8)	24 (9)	1.2 (0.77, 1.8)	12 (9)	1.3 (0.68, 2.3)
> 18 pack-years	1,388 (6)	29 (11)	1.5 (1.0, 2.2)^*^	13 (10)	1.5 (0.84, 2.7)
Grew up on farm
No	9,202 (39)	94 (35)	1.0 (referent)	47 (37)	1.0 (referent)
Yes	14,196 (61)	172 (65)	0.94 (0.73, 1.2)	80 (63)	0.93 (0.64, 1.3)
Years on farm
< 11 years	3,168 (14)	15 (6)	1.0 (referent)	10 (8)	1.0 (referent)
11–20 years	3,936 (17)	36 (13)	1.6 (0.88, 3.0)	18 (14)	1.3 (0.60, 2.9)
21–30 years	4,237 (18)	53 (20)	1.8 (0.99, 3.2)	24 (19)	1.3 (0.62, 2.9)
≥ 31 years	11,986 (51)	163 (61)	1.3 (0.75, 2.3)	74 (59)	1.1 (0.55, 2.3)
Note: ORs and 95% CIs for all RA cases and incident cases were adjusted for age. ^*^p < 0.05.					

### Pesticides

RA cases were slightly more likely to report any lifetime pesticide use (Table 2), but there was no evidence of an exposure response for years or days per year pesticides were applied and for indicators of higher personal exposures (e.g., mixing at least 50% of the time). RA was associated with reported use of any specified pesticides. Of 15 pesticides examined, maneb/mancozeb was associated with RA overall and with incident RA. Incident RA was also associated with glyphosate. No statistically significant associations were seen for non-specified residential pesticides or pesticide used or chemical classes (e.g., insecticides or organochlorines; not shown). Adjusting for the five most common pesticides (carbaryl, diazinon, glyphosate, malathion, and 2,4-D), incident RA remained associated with glyphosate (OR = 1.4; 95% CI: 1.0, 2.1).

**Table 2:** Specific pesticides associated with RA, in all cases and incident cases for female spouses in the Agricultural Health Study.

Lifetime pesticide use	Comparison *n *= 23,570 *n* (%)	All RA cases		Incident cases	
		Case *n *= 271 *n *(%)	OR (95%CI)	Case *n *= 129 *n* (%)	OR (95%CI)
Ever mix or apply pesticides
No	9,924 (42)	103 (38)	1.0 (referent)	44 (34)	1.0 (referent)
Yes	13,645 (58)	168 (62)	1.2 (0.97, 1.6)	85 (66)	1.4 (0.99, 2.1)
Years applied pesticides^*a*^
≤ 20	10,881 (46)	119 (44)	1.2 (0.90, 1.6)	65 (50)	1.5 (0.98, 2.2)
> 20	2,764 (12)	49 (18)	1.2 (0.90, 2.0)	20 (16)	1.3 (0.70, 2.5)
Days per year applied^*a*^
< 20	11,631 (49)	142 (52)	1.2 (0.95, 1.6)	70 (54)	1.4 (0.94, 2.1)
≥ 20	2,014 (9)	26 (10)	1.2 (0.74, 2.1)	15 (12)	1.6 (0.83, 3.2)
Percent of time applied^*a*^
Did not apply	959 (4)	9 (3)	0.94 (0.46, 1.9)	6 (5)	1.3 (0.49, 3.7)
< 50% of time	6,934 (30)	89 (33)	1.3 (0.95, 1.7)	46 (35)	1.5 (0.98, 2.3)
≥ 50% of time	5,752 (24)	70 (26)	1.2 (0.89, 1.7)	33 (26)	1.3 (0.81, 2.2)
Percent of the time mixed^*a*^
Did not mix	5,100 (22)	58 (21)	1.2 (0.83, 1.6)	30 (23)	1.4 (0.86, 2.3)
< 50% of time	5,494 (23)	67 (25)	1.2 (0.88, 1.7)	37 (29)	1.5 (0.97, 2.4)
≥ 50% of time	3,051 (13)	43 (16)	1.4 (0.94, 2.0)	18 (14)	1.3 (0.73, 2.3)
Types of pesticides^*b*^
None reported	7,840 (33)	76 (28)	1.0 (referent)	36 (28)	1.0 (referent)
Not specified	3,145 (13)	38 (14)	1.3 (0.90, 2.0)	15 (12)	1.1 (0.59, 2.0)
Any specified	12,585 (53)	157 (58)	1.4 (1.0, 1.8)*	78 (60)	1.4 (0.93, 2.1)
Glyphosate	8,140 (35)	100 (38)	1.2 (0.95, 1.6)	54 (43)	1.4 (1.0, 2.1)*
Carbaryl	7,421 (32)	94 (36)	1.1 (0.85, 1.4)	41 (33)	0.98 (0.67, 1.4)
Malathion	4,671 (20)	58 (22)	1.1 (0.80, 1.4)	23 (19)	0.86 (0.55, 1.4)
2,4-D	3,536 (15)	31 (12)	0.75 (0.51, 1.1)	14 (11)	0.69 (0.39, 1.2)
Diazinon	2,448 (11)	34 (13)	1.2 (0.83, 1.7)	15 (12)	1.1 (0.67, 2.0)
Trifluralin	1,245 (5)	8 (3)	0.57 (0.28, 1.1)	3 (2)	NC (—)
Atrazine	1,050 (5)	8 (3)	0.65 (0.32, 1.3)	4 (3)	NC (—)
DDT	806 (4)	17 (7)	1.5 (0.89, 2.4)	10 (8)	1.9 (0.97, 3.6)
Permethrin (animals)	849 (4)	12 (5)	1.5 (0.83, 2.7)	7 (6)	1.7 (0.80, 3.7)
Chlordane	984 (4)	12 (5)	0.99 (0.57, 1.7)	6 (5)	1.0 (0.44, 2.3)
Dicamba	949 (4)	7 (3)	0.68 (0.32, 1.5)	4 (3)	NC (—)
Imazethapyr	696 (3)	8 (3)	1.1 (0.55, 2.3)	4 (3)	NC (—)
Dichlorvos	618 (3)	8 (3)	1.1 (0.56, 2.4)	4 (3)	NC (—)
Captan	541 (2)	5 (2)	0.75 (0.31, 1.8)	5 (4)	1.6 (0.66, 4.0)
Maneb/Mancozeb	351 (1)	10 (4)	2.0 (1.1, 3.9)	7 (6)	3.3 (1.5, 7.1)
Note: ORs and 95% CIs adjusted for age, state and pack-years smoking; only for pesticides or classes with at least 5 incident cases exposed. Women who did not provide any data on use of pesticides at Phase 1 were excluded from this table (452 female spouses, including 4 cases). ^***a***^Missing data on frequency or duration imputed for 3,413 (14%) of noncases and 41 cases (15%); referent did not mix or apply any pesticides. ^***b***^Not specified include residential pesticides; specific pesticide classes represented include herbicides (glyphosate, 2,4-D, trifuralin, atrazine, dicamba, imazethapyr), insecticides (carbaryl, malathion, diazinon, DDT, permethrin, clordane, dichlorvos), and fungicides (captan, maneb/mencozeb). ^*^p < 0.05.					

In sensitivity analyses of cases diagnosed after the first 2 years of follow-up (*n* = 96), we confirmed associations of incident RA with any specified pesticide use (OR = 1.6; 95% CI: 1.0, 2.4) and maneb/mancozeb (OR = 3.2; 95% CI: 1.3, 8.1), while the association was similar for glyphosate, though no longer statistically significant (OR = 1.4; 95% CI: 0.9, 2.1). Including possible cases and limiting to those reporting a positive RF-test (*n* = 174 total, 69 incident), the association with incident RA was similar for maneb/mancozeb (OR = 3.9; 95% CI: 1.5, 10.1), increased for dichlorodiphenyltrichloroethane (DDT) (OR = 2.8; 95% CI: 1.2, 6.3), and decreased for glyphosate (OR = 1.3; 95% CI: 0.8, 2.1).

### Other Exposures

Incident RA cases were somewhat more likely than noncases to report no field work in the growing season prior to enrollment (55% versus 48%) (Table 3). However, incident RA was positively associated with the use of chemical fertilizers and solvents in the past season at enrollment, and inversely associated with livestock exposure both as a child and as an adult compared with no exposure. We saw no confounding by the five most common pesticides or childhood animal exposures (not shown). Excluding cases diagnosed in the first 2 years of follow-up, associations with solvents (OR = 1.5; 95% CI: 0.95, 2.5) and chemical fertilizers (OR = 1.4; 95% CI: 0.8, 2.6) were slightly reduced. Adding “possible” cases and limiting by RF status, associations with solvents, chemical fertilizers, and livestock exposure were similar to those in Table 3 (not shown).

**Table 3:** Field work and other farm exposures in relation to incident RA cases for female spouses in the Agricultural Health Study.

Tasks and other farm exposures	Incident cases		
	Noncases *n*= 24,018 *n *(%)	Cases *n *= 132 *n* (%)	OR (95% CI)^*a*^
Worked in the field recent growing season
No	11,132 (48)	71 (55)	1.0 (referent)
< 10 days	4,766 (20)	22 (17)	0.78 (0.48, 1.3)
10–30 days	4,183 (18)	23 (18)	0.89 (0.55, 1.4)
> 30 days	3,235 (14)	13 (10)	0.63 (0.35, 1.4)
Tasks^*b*^
Till soil	5,740 (25)	27 (22)	0.88 (0.57, 1.4)
Planting	5,538 (24)	40 (32)	1.3 (0.91, 2.0)
Apply natural fertilizer	2,700 (12)	13 (11)	0.85 (0.48, 1.5)
Apply chemical fertilizer	2,540 (11)	23 (18)	1.7 (1.1, 2.7)
Drive combines	2,545 (11)	11 (9)	0.86 (0.46, 1.6)
Handpick crops	5,909 (25)	37 (29)	1.1 (0.75, 1.7)
Other tasks, at least monthly^*b*^
Drive trucks	8,942 (39)	42 (35)	0.88 (0.59, 1.3)
Drive diesel tractor	7,727 (34)	33 (27)	0.77 (0.49, 1.1)
Drive gas tractor	5,956 (26)	32 (26)	1.0 (0.65, 1.5)
Clean with gasoline	3,440 (15)	18 (15)	1.0 (0.61, 1.7)
Clean with solvents	4,522 (20)	33 (27)	1.6 (1.1, 2.4)
Painting	7,246 (32)	45 (37)	1.3 (0.91, 1.9)
Veterinary procedures	2,927 (13)	13 (11)	0.99 (0.55, 1.8)
Hours per day in the sun, recent growing season
< 1	4,678 (26)	32 (29)	1.0 (referent)
1 to 2	5,769 (33)	42 (39)	1.1 (0.72, 1.8)
3 to 5	5,047 (29)	26 (24)	0.82 (0.49, 1.4)
≥ 6	2,127 (12)	9 (8)	0.67 (0.32, 1.4)
Hours per day in the sun, 10 years ago
< 1	2,940 (18)	13 (14)	1.0 (referent)
1 to 2	4,391 (26)	30 (28)	1.4 (0.75, 2.6)
3 to 5	5,724 (35)	44 (42)	1.6 (0.86, 2.8)
≥ 6	3,506 (21)	16 (15)	0.89 (0.44, 1.8)
Regular livestock contact, past 12 months^*c*^
Never	10,820 (46)	60 (54)	1.0 (referent)
Infrequent	6,765 (29)	35 (27)	0.99 (0.65, 1.5)
Frequent	5,699 (24)	24 (19)	0.82 (0.51, 1.3)
Livestock exposure, in childhood^*c*^
None	4,752 (27)	33 (29)	1.0 (referent)
Infrequent	2,469 (14)	13 (11)	0.81 (0.42, 1.6)
Frequent	10,589 (59)	68 (60)	0.87 (0.57, 1.3)
Any childhood and current livestock exposure
Neither	3,754 (21)	28 (25)	1.0 (referent)
Childhood only	9,256 (52)	70 (61)	0.96 (0.62, 1.5)
Current only	998 (6)	5 (4)	0.84 (0.32, 2.2)
Childhood and current	3,802 (21)	11 (10)	0.48 (0.24, 0.97)
^***a***^Logistic regression models adjusted for age, state and pack-years smoking, estimated ORs and 95% CI. ^***b***^Referent group was an answer of “no” for each task. ^***c***^Infrequent is less than once per week, and frequent is at least weekly.			

### Subgroup Analyses

**Figure 1:**
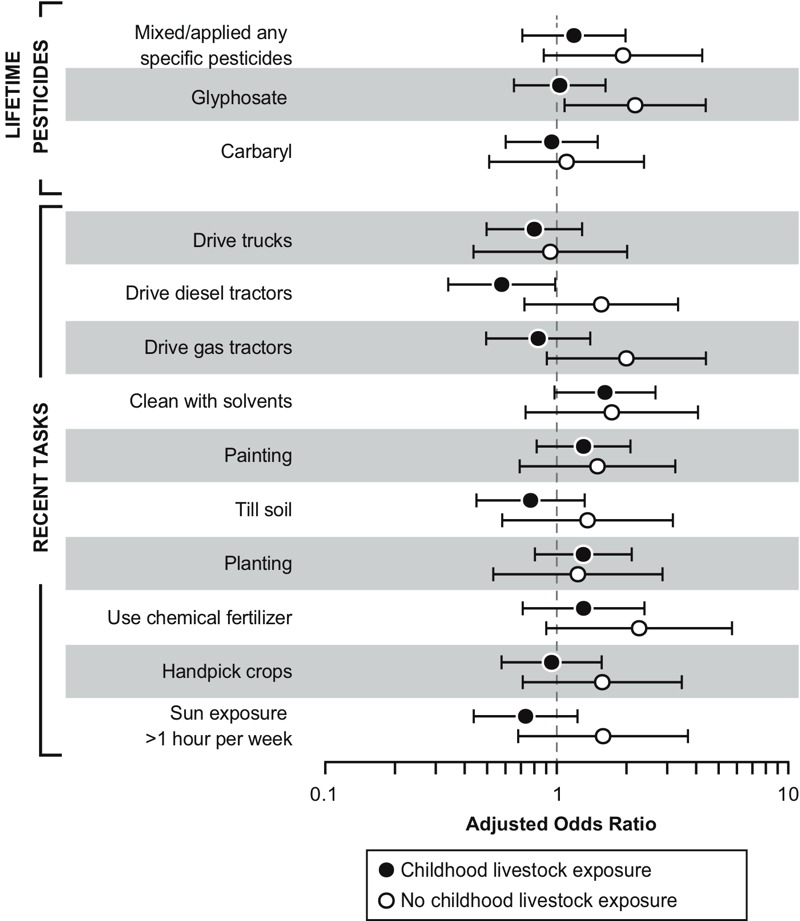
Incident RA associations with pesticides and farm tasks, stratified by childhood livestock exposure. Note: Analyses limited to specified pesticides with at least 5 exposed cases.

Stratified models revealed qualitative differences in associations with incident RA, depending on whether women had lived at least half their life prior to age 18 on a farm (“childhood farm residence”; see Table S2). In women with a childhood farm residence, we observed associations with DDT (OR = 2.0; 95% CI: 1.0, 4.2) and maneb/mancozeb (OR = 3.7; 95% CI: 1.6, 8.8), while these exposures were rare among women without a childhood farm residence. Among women without a childhood farm residence, incident RA was associated with glyphosate (OR = 1.9; 95% CI: 1.1, 3.4). Interactions were not statistically significant (*p* > 0.10). The frequency of field work and many tasks in the past growing season was higher among women with a childhood residential farm history, and childhood livestock exposure was much more common (e.g., contact at least once per week was reported by 90% of incident cases vs. 8% of those without a childhood farm residence). We explored RA risk factors stratified by childhood livestock exposure as a more direct indicator of potential immune modifying exposures (Figure 1). Associations of incident RA with glyphosate were significant only in women *without* childhood livestock exposure, and associations with several other (but not all) farm tasks appeared to vary by livestock exposure.

State-stratified analyses suggested differences in RA risk associated with any use of specified pesticides (e.g., in NC, OR = 2.4; 95% CI: CI 1.2, 4.7; see Table S3), and with DDT (OR = 4.7, 95% CI: 2.0, 10.8).

## Discussion

In this study of female spouses of licensed pesticide applicators, RA was associated with reporting any specified pesticides, and some individual associations were notable. Use of maneb/mancozeb was positively associated both with overall and incident RA. Manganese-containing carbamate fungicides, maneb or mancozeb were also associated with thyroid disease in AHS spouses (Goldner et al. 2010). This is the first study to link maneb/mancozeb to a systemic autoimmune disease; the finding was robust, but only 4% of cases were exposed. The most commonly used herbicide, glyphosate, was modestly associated with RA risk. Glyphosate has been inconsistently associated with non-Hodgkin lymphoma (Schinasi and Leon 2014), but has not previously been associated with RA. Further studies are warranted due to the extensive use of glyphosate in agricultural and residential settings. We also noted an elevated, non-statistically significant association of DDT with incident RA.

Use of chemical fertilizers and solvents were also associated with RA risk. Chemical fertilizers are concentrated formulations of common ingredients, including nitrogen, phosphorus, and potassium, and may be contaminated with metals such as uranium and arsenic (Schnug and Lottermoser 2013). Field work, fertilizer use and other tasks have been generally associated with use of more pesticides in AHS spouses (Kirrane et al. 2004), but specific pesticides were not correlated with chemical fertilizer use in this sample. Occupational solvent use has been associated with several autoimmune diseases, but evidence is limited for RA (Barragán-Martínez et al. 2012).

An inverse association between RA risk and the combined childhood and adult contact with livestock suggests protective factors related to animal exposures across the lifespan. This result is consistent with a prior study of SLE (Parks et al. 2008), but to our knowledge has not been previously described for RA. Early-life exposures to infections, the microbiome, and microbial products, such as endotoxin, are known to play an important role in programming immune system responses in later life (Morris et al. 2015; von Mutius and Vercelli 2010). Our findings may explain prior studies showing a lack of a positive association of RA with livestock farming (Gold et al. 2007; Lee et al. 2002). Farmers are often raised in an agricultural setting, with frequent and prolonged exposures to farm animals in early life, potentially protecting against the development of immune dysregulation and associated diseases in adulthood (Rook 2012). Although the role of microbial exposures on autoimmune disease etiology is complex (Bach 2005), our results point towards a need for further investigation of early life exposures to infections and other organic products.

Mechanisms by which pesticides might influence development RA are diverse. The robust association of RA with maneb/mancozeb is supported by a small body of experimental research on maneb immunotoxicity *in vitro* (Mandarapu et al. 2014; Mandarapu and Prakhya 2015) and *in vivo* (Chung and Pyo 2005). Acute maneb immunotoxic effects in humans are thought to be limited (Corsini et al. 2013), but chronic exposure has been associated with leukemia in a study of farm workers (Mills et al. 2005). The link between DDT and autoimmunity is the most developed of all the pesticides evaluated in this study. Although DDT has immune suppressive effects (Corsini et al. 2013), the metabolite dichlorodiphenyldichloroethylene (DDE) can induce both apoptosis and inflammation in peripheral blood mononuclear cells (Alegría-Torres et al. 2009). In a representative sample of the U.S. population, self-reported RA cases had higher levels of organochlorine pesticides, including DDE, than did noncases (Lee et al. 2007). Glyphosate effects on the immune system are not well known, though there is evidence it can induce pulmonary inflammation and cytokines associated with a Th2 immune response (Kumar et al. 2014). An intriguing, albeit indirect, pathway linking glyphosate with RA could be the release of remnant DDT in contaminated soils (Sabatier et al. 2014), perhaps through erosion and soil dust exposure. Soil dust is also a potential source of silica exposure (Swanepoel et al. 2010), an established risk factor for RA (Miller et al. 2012).

Many pesticides may act through endocrine pathways, including maneb/mancozeb (Axelstad et al. 2011; Bisson and Hontela 2002), DDT (McKinlay et al. 2008), and glyphosate-containing products (Gasnier et al. 2009; Richard et al. 2005). Some previous studies reported an RA–farming association seen primarily in men (Lee et al. 2002; Li et al. 2008; Lundberg et al. 1994; Milham 1988; Olsson et al. 2000). This may reflect a scarcity of women reporting farming occupation, sex-differences in tasks and exposures, or potential modifying effects of hormones or other factors. We did not see confounding by hormone use and or notable differences in pesticide associations by menopause status at diagnosis (not shown).

This study has several strengths including data on specific pesticides and agricultural exposures. While this is the largest study to date of specific agricultural pesticide use and RA, our analysis of pesticides is still limited by exposure misclassification, with no information on the timing or frequency of specific pesticide use. We did not consider para-occupational exposures and agricultural drift, which may contribute to elevated background exposures even in women who did not apply pesticides themselves (Deziel et al. 2015). Due to a high proportion of missing data, we evaluated overall pesticide dose–response associations after imputing data that were missing. Performing multiple imputation assumes data are missing at random, which cannot be proven. However, our results were similar in a complete case analysis (not shown). Besides a lack of dose-data on specific pesticides, reasons for the lack of an apparent overall dose–response could include unmeasured factors, such as added exposures due to concurrent off-farm job responsibilities. Many women were exposed to more than one pesticide and other farm characteristics. However, we did not specifically seek to explore risks due to multiple pesticides or combinations of specific pesticides due to the relatively small case sample. Due to the low prevalence and weak correlation of maneb use with other pesticides, we did not consider maneb in combination with either glyphostate or DDT. Moreover, in post hoc analyses we saw no evidence of a stronger association with RA in women who used both DDT and glyphosate (not shown).

Many female spouses in the AHS participated in farming activities and may have had exposures within the range of some farmers who apply pesticides (Kirrane et al. 2004). Only two-thirds of the women had a long-term childhood farm history, which enabled us to examine heterogeneity by earlier-life farm environment and livestock exposure. In a prior analysis of AHS female spouses, a modest interaction was seen between childhood farm residence and any pesticide use for atopic asthma (Hoppin et al. 2008). Besides livestock exposure, other factors are likely to differ by childhood farm residence, for example, potential for early or prolonged pesticide exposures.

In addition to physician-confirmed cases, we used screening data to identify probable “clinical” cases confirmed by self-reported use of RA-specific medications. This gave us more power to focus on incident cases, which may minimize the influence of recall bias or healthy worker effect. Confirming RA cases based on reported DMARD use is a relatively cost-effective method to identify cases with high specificity (Walitt et al. 2008), but may be insensitive to cases with less active or severe disease or those who have less access to or choose not to use these medications. Adding possible cases (treated with corticosteroids only) increased the proportion of cases from NC. Associations were similar in this larger, more inclusive case group (not shown), while limiting to a more specific phenotype (i.e., RF-positive cases) yielded a stronger association with DDT. Incomplete case ascertainment is a concern due to loss of statistical power and potential selection bias. Among the 1,026 women with self-reported RA deemed ineligible for screening (e.g., missing age at diagnosis or inconsistent reporting), linkage to vital records identified four deaths with RA (0.39%). We considered inconsistent reporting (i.e., “yes” at either Phase 1 or 2 and then “no” at a later phase) the same as refuting a diagnosis in our screening calls. Those ineligible for screening tended to be older and from NC, both risk factors for RA. Although the small number of missing cases is unlikely to have biased our findings, their omission may fail to represent some women with higher DDT exposure, for example. Noncases in NC were less likely to report pesticides and livestock contact compared to their counterparts in IA, but they were more likely to report planting, use of chemical fertilizers and picking crops. Some differences in pesticide associations by state could, therefore, reflect differences in the type of direct (e.g., handpicking) or protective (e.g., livestock) co-exposures. Differences could be due to chance. However, we did not adjust for multiple comparisons through methods such as Bonferroni-type correction, given our strong prior rationale on pesticides and farming, including the 15 specific pesticides (out of 50 possible) and 18 farm tasks and exposures examined, preferring to present estimated associations, confidence limits and statistical testing in the context of prior evidence, potential biases, strengths and limitations of the study design, and available data (Goldberg and Silbergeld 2011).

## Conclusion

Leveraging the detailed exposure data and longitudinal design of the AHS, our results provide new evidence linking specific pesticides and other types of farm exposures with risk of RA. Several potential risk factors were identified, but our findings also suggest the importance of considering both protective and risk-related exposures across the life course. To generalize findings, replication is warranted in other agricultural and population settings.

**Editor’s Note:** In the Study Sample section of the Methods, the Advance Publication incorrectly indicated that 123 women who were excluded were eligible for screening in the prior study. The sentence should have indicated that they were ineligible for screening in the prior study. The error has been corrected in this version of the article.

## Supplemental Material

(434 KB) PDF
