# Supplementary material for: Rheumatoid Arthritis in Agricultural Health Study Spouses: Associations with Pesticides and Other Farm Exposures
Source: Environ Health Perspect. 2016 Jun 10;124(11):1728–34. doi: 10.1289/EHP129 (PMC5089872; doi:10.1289/EHP129)
Supplement: (434 KB) PDF [file EHP129.s001.acco.pdf]

Environ Health Perspect
DOI: 10.1289/EHP129

Note to readers with disabilities: *EHP* strives to ensure that all journal content is accessible to all readers. However, some figures and Supplemental Material published in *EHP* articles may not conform to 508 standards due to the complexity of the information being presented. If you need assistance accessing journal content, please contact ehp508@niehs.nih.gov. Our staff will work with you to assess and meet your accessibility needs within 3 working days.

Supplemental Material

Rheumatoid Arthritis in Agricultural Health Study Spouses: Associations with Pesticides and Other Farm Exposures

Christine G. Parks, Jane A. Hoppin, Anneclaire J. DeRoos, Karen H. Costenbader, Michael C. Alavanja, and Dale P. Sandler

Table of Contents

Figure S1. Identification of RA cases and non-cases
Table S1. Characteristics of physician confirmed and probable RA cases included in analyses, and other self-reported RA cases excluded from analyses
Table S2. Risk of incident RA associated with recent farm exposures, stratified by childhood farm residence
Table S3. Risk of RA associated with specific pesticides, stratified by state
Reference

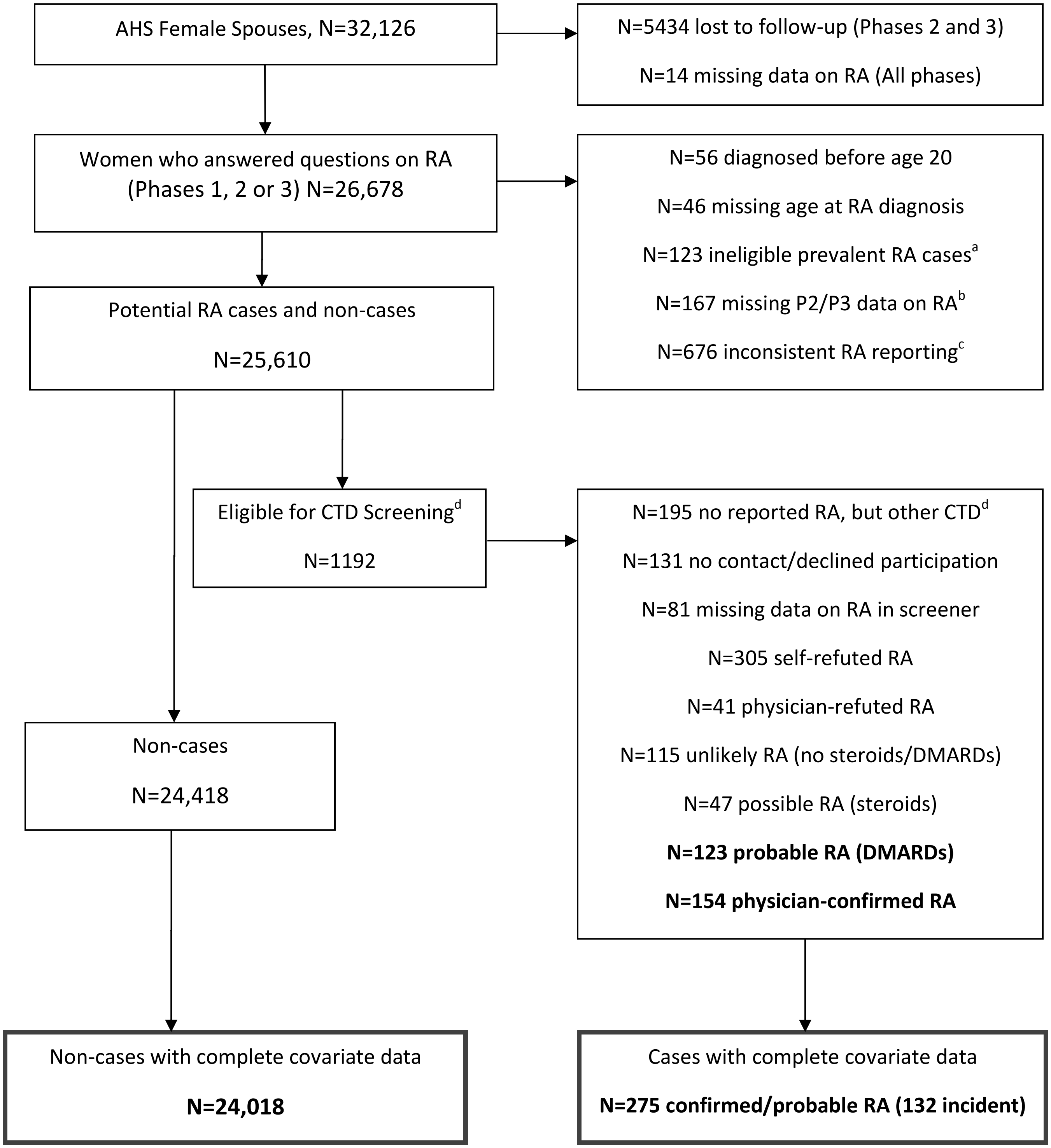

Figure S1. Identification of RA cases and non-cases

[a]Prevalent cases not included in prior study (DeRoos AJ et al., 2005)

[b]Women who completed AHS follow-up questionnaires, but did not provide responses on RA status

[c]Initial self-report not confirmed by in subsequent AHS questionnaires

[e]Eligibility based on inclusion in prior study (mostly prevalent RA; De Roos AJ et al., 2005) and all new incident RA cases (total N=795), and those with related connective tissue diseases (CTD; systemic lupus erythematosus and Sjögren’s syndrome – SLE and SS)

Table S1. Characteristics of physician confirmed and probable RA cases included in analyses, and other self-reported RA cases excluded from analyses

	Confirmed/probable RA				Self-reported RA, excluded[a]					
	Physician Confirmed N=154		DMARD use/ Probable N=123		Steroid use/ Possible N=47		No DMARD or steroid use N=115		Physician Refuted N=41	
	N	%	N	%	N	%	N	%	N	%
Age (years)										
<40	20	*13*	15	*12*	4	*8*	22	*19*	3	*7*
40-49	33	*21*	33	*27*	8	*17*	24	*21*	11	*27*
50-59	57	*37*	50	*41*	21	*45*	39	*34*	18	*44*
60+	44	*29*	25	*20*	14	*30*	30	*26*	9	*22*
State										
NC	48	*31*	57	*46*	26	*55*	44	*38*	12	*29*
IA	106	*69*	66	*54*	21	*45*	71	*62*	29	*71*
Diagnosis age										
16 to 42	59	*38*	32	*26*	10	*24*	38	*34*	20	*50*
43 to 51	34	*22*	30	*25*	15	*35*	33	*30*	8	*20*
52 to 59	32	*21*	28	*23*	7	*17*	23	*21*	5	*13*
60 to 82	29	*19*	32	*26*	10	*24*	17	*15*	7	*18*
Missing	0	--	1	--	5	--	4	--	1	--
Diagnosis year[b]										
Before 1990	69	*45*	32	*26*	16	*38*	42	*38*	16	*40*
1990 to 1999	68	*44*	38	*31*	16	*38*	49	*44*	22	*50*
2000 and later	17	*11*	52	*43*	10	*24*	20	*18*	4	*10*
Missing	0	--	1	--	5	--	4	--	1	--
Rheumatologist[c]										
Never Seen	13	*10*	11	*9*	21	*46*	57	*71*	19	*66*
Seen By	9	*7*	16	*13*	3	*7*	7	*9*	4	*14*
Diagnosed By	106	*83*	94	*78*	22	*48*	16	*20*	6	*21*
Don't know	2	--	2	--	1	--	2	--	0	--
Joint symptoms 6+ weeks[c]										
No	12	*8*	14	*13*	10	*21*	15	*23*	1	*3*
Yes	139	*92*	98	*87*	37	*79*	51	*77*	30	*97*
RF test[c]										
Not tested	6	*5*	14	*15*	20	*51*	28	*43*	12	*39*
Tested negative	9	*8*	6	*6*	1	*3*	2	*3*	1	*3*
Tested positive	90	*81*	70	*75*	16	*41*	20	*46*	12	*39*
Don't know result	6	*5*	3	*3*	2	*5*	7	*10*	3	*10*
Don't know if tested	18	--	19	--	9	--	9	--	3	--

[a]Total of 784 were screened (784 were screened for RA plus 7 were screened for another CTD and reported RA for the first time on the screener); of those screened, 305 refuted their diagnosis. Of categories shown, unconfirmed = reported no DMARD or steroid use, possible = reported steroid use for RA, probable = reported DMARD use for RA; physician-refuted and confirmed cases were identified based on a subset with validation data.

[b]Diagnosis year could not be calculated for those who reported an age-range of diagnosis.

[c]Questions not administered to 29 women who completed a mailed screener (13 self-refuted, 5 unconfirmed and 11probable cases). Due to changes in the screening protocol, others were not asked about seeing a rheumatologist (33 unconfirmed, 25 physician confirmed, and 12 physicians refuted). RF and symptom data were not collected from many, but not all of these same cases (not asked: 49 unconfirmed, 10 probable, 3 confirmed and 12 refuted by physician).

Table S2. Risk of incident RA associated with recent farm exposures, stratified by childhood farm residence

	No childhood farm			Yes childhood farm		
	Comparison N=9202 %	Case N=47 %	OR95%CI[a]	Comparison N=14,197 %	Case N=80 %	OR95%CI[a]
**Lifetime use of pesticides**						
None reported	*39*	*34*	1.0 (referent)	*29*	*24*	1.0 (referent)
Not specified	*15*	*4*	NC (---)	*12*	*15*	1.6 (0.81, 1.3)
Any specified	*47*	*62*	1.5 (0.80, 2.8)	*58*	*61*	1.4 (0.86, 1.8)
Glyphosate	*31*	*46*	1.9 (1.1, 3.4)	*38*	*41*	1.2 (0.73, 1.4)
Carbaryl	*26*	*32*	1.3 (0.69, 2.4)	*36*	*34*	0.86 (0.71, 1.3)
Malathion	*16*	*15*	0.90 (0.40, 2.0)	*23*	*21*	0.96 (0.67, 1.4)
2, 4-D	*11*	*11*	0.92 (0.36, 2.4)	*18*	*12*	0.60 (0.29, 1.2)
Diazinon	*9*	*9*	NC (---)	*12*	*15*	1.3 (0.64, 2.3)
DDT	*2*	*2*	NC (---)	*5*	*12*	2.0 (1.0, 4.2)[#]
Maneb	*1*	*2*	NC (---)	*2*	*8*	3.7 (1.6, 8.8)
**Current growing season, Field work**						
None	*55*	*60*	1.0 (referent)	*43*	*53*	1.0 (referent)
Less than 10 days	*20*	*17*	0.81 (0.37, 1.9)	*21*	*17*	0.79 (0.43, 1.4)
10–30 days	*15*	*13*	0.80 (0.33, 1.9)	*20*	*21*	0.93 (0.53, 1.6)
More than 30 days	*10*	*11*	1.0 (0.35, 2.6)	*16*	*9*	0.45 (0.20, 1.1)
**Specific tasks**						
Till the soil	*19*	*20*	1.1 (0.52, 2.3)	*28*	*22*	0.76 (0.44, 1.3)
Planting	*20*	*26*	1.3 (0.65, 2.6)	*26*	*34*	1.3 (0.80, 2.1)
Use natural fertilizer	*9*	*9*	NC (---)	*13*	*10*	0.71 (0.34, 1.5)
Use chemical fertilizer	*9*	*16*	1.8 (0.78, 4.1)	*12*	*19*	1.5 (0.85, 2.7)
Drive combined	*9*	*9*	NC (---)	*13*	*8*	0.64 (0.27, 1.5)
Hand-pick crops	*23*	*31*	1.5 (0.78, 2.9)	*27*	*29*	0.97 (0.58, 1.6)
**Other regular tasks past 12 months**						
Drive trucks	*37*	*28*	0.67 (0.34, 1.3)	*41*	*39*	1.0 (0.65, 1.6)

Use a diesel tractor	*27*	*29*	1.2 (0.61, 2.2)	*38*	*26*	0.63 (0.37, 1.1)
Use a gas tractor	*20*	*27*	1.5 (0.79, 3.0)	*30*	*26*	0.84 (0.50, 1.4)
Clean with gas	*11*	*9*	NC (---)	*18*	*18*	1.1 (0.62, 2.0)
Clean with solvents	*17*	*23*	1.5 (0.70, 2.9)	*22*	*30*	1.7 (1.0, 2.8)
Painting	*29*	*30*	1.1 (0.55, 2.1)	*34*	*41*	1.5 (0.96, 2.4)
Veterinary procedures	*10*	*10*	NC (---)	*15*	*12*	0.98 (0.48, 2.0)
**Hours per day in the sun, recent growing season**						
< 1	*30*	*26*	1.0 (referent)	*25*	*31*	1.0 (referent)
1 to 2	*34*	*37*	1.3 (0.61, 2.9)	*32*	*40*	1.1 (0.60, 1.9)
3 to 5	*27*	*26*	1.2 (0.51, 2.8)	*30*	*23*	0.68 (0.35, 1.3)
6+	*10*	*12*	1.5 (0.51, 4.3)	*14*	*6*	0.41 (0.14, 1.2)
**Hours per day in the sun, 10 years ago**						
< 1	*21*	*15*	1.0 (referent)	*16*	*14*	1.0 (referent)
1 to 2	*28*	*22*	1.2 (0.42, 3.3)	*25*	*31*	1.4 (0.64, 3.1)
3 to 5	*33*	*48*	2.1 (0.84, 5.4)	*36*	39	1.3 (0.58, 2.7)
6+	*17*	*15*	1.3 (0.41, 4.0)	*23*	*15*	0.70 (0.29, 1.8)
**Livestock contact past year**						
Never	*52*	*53*	1.0 (referent)	*43*	*54*	1.0 (referent)
< 1X/week	*29*	*26*	0.96 (0.48, 2.0)	*29*	*29*	1.0 (0.53, 1.7)
≥1X per week	*19*	*21*	1.2 (0.56, 2.6)	*28*	*17*	0.67 (0.34, 1.3)
**Livestock contact, childhood**						
Never	*59*	*71*	1.0 (referent)	*6*	*4*	1.0 (referent)
< 1X/week	*24*	*21*	0.78 (0.35, 1.7)	*8*	*6*	NC (---)
≥1X per week	*17*	*8*	NC (---)	*86*	*90*	NC (---)

[#]P<0.05

[a]Odds Ratio (OR) and 95% Confidence Interval (CI) adjusted for age, smoking pack years

Table S3. Risk of RA associated with specific pesticides, stratified by state

	North Carolina			Iowa		
	Comparison N=6,929 %	Case N=46 %	OR(95%CI)[a]	Comparison N=17,089 %	Case N=86 %	OR(95%CI)[a]
**Lifetime pesticide use**						
None	*43*	*27*	1.0 (referent)	*29*	*28*	1.0 (referent)
Not specified	*10*	*5*	0.7 (0.15, 3.1)	*14*	*15*	1.1 (0.54, 2.1)
Any specified	*46*	*68*	2.4 (1.2, 4.7)	*56*	*56*	0.99 (0.61, 1.6)
Glyphosate	*29*	*40*	1.7 (0.91, 3.1)	*37*	*44*	1.3 (0.86, 2.0)
Carbaryl	*34*	*44*	1.5 (0.82, 2.7)	*31*	*27*	0.74 (0.46, 1.2)
Malathion	*17*	*21*	1.3 (0.61, 2.7)	*21*	*17*	0.70 (0.39, 1.2)
Diazinon	*13*	*22*	2.0 (0.93, 4.1)	*9*	*7*	0.71 (0.31, 1.6)
2, 4 D	*6*	*0*	NC (---)	*19*	*17*	0.81 (0.46, 1.4)
Chlordane	*3*	*1*	NC (---)	*5*	*6*	1.1 (0.45, 2.8)
Permethrin (animals)	*2*	*5*	NC (---)	*5*	*6*	1.4 (0.58, 3.6)
DDT	*4*	*17*	4.7 (2.0, 10.8)	*1*	*4*	NC

[a]Odds Ratio (OR) and 95% Confidence Interval (CI) adjusted for age, smoking pack years.

REFERENCE

De Roos AJ, Cooper GS, Alavanja MC, et al. Rheumatoid arthritis among women in the Agricultural Health Study: risk associated with farming activities and exposures. Ann Epidemiol. 2005 Nov;**15**:762-70.
